# Cell death-related molecules and biomarkers for renal cell carcinoma targeted therapy

**DOI:** 10.1186/s12935-019-0939-2

**Published:** 2019-08-23

**Authors:** Yongchang Lai, Tao Zeng, Xiongfa Liang, Weizou Wu, Fangling Zhong, Wenqi Wu

**Affiliations:** Department of Urology, Minimally Invasive Surgery Center, Guangdong Key Laboratory of Urology, Guangzhou Urology Research Institute, The First Affiliated Hospital of Guangzhou Medical University, Kangda Road 1#, Haizhu District, Guangzhou, 510230 Guangdong China

**Keywords:** Cell death, Targeted therapy, Renal cell carcinoma, NF-κB, Apoptosis

## Abstract

Renal cell carcinoma (RCC) is not sensitive to conventional radio- and chemotherapies and is at least partially resistant to impairments in cell death-related signaling pathways. The hallmarks of RCC formation include diverse signaling pathways, such as maintenance of proliferation, cell death resistance, angiogenesis induction, immune destruction avoidance, and DNA repair. RCC diagnosed during the early stage has the possibility of cure with surgery. For metastatic RCC (mRCC), molecular targeted therapy, especially antiangiogenic therapy (e.g., tyrosine kinase inhibitors, TKIs, such as sunitinib), is one of the main partially effective therapeutics. Various forms of cell death that may be associated with the resistance to targeted therapy because of the crosstalk between targeted therapy and cell death resistance pathways were originally defined and differentiated into apoptosis, necroptosis, pyroptosis, ferroptosis and autophagic cell death based on cellular morphology. Particularly, as a new form of cell death, T cell-induced cell death by immune checkpoint inhibitors expands the treatment options beyond the current targeted therapy. Here, we provide an overview of cell death-related molecules and biomarkers for the progression, prognosis and treatment of mRCC by targeted therapy, with a focus on apoptosis and T cell-induced cell death, as well as other forms of cell death.

## Background

Renal cell carcinoma (RCC) is characterized by uncontrolled cell proliferation and an absence of cell death and is not sensitive to conventional radio- and chemotherapies and is at least partly resistant to impairments in both extrinsic and intrinsic apoptotic pathways [1]. The hallmarks of tumor formation include diverse signaling pathways, such as maintenance of proliferation, cell death resistance, angiogenesis induction, immune destruction avoidance, and DNA repair [2]. Poor selectivity, strong side effects and drug resistance are the main barriers for chemotherapeutic drugs. Early-stage RCC has the possibility of cure by resection, while targeted therapy is recommended for metastatic RCC (mRCC). Targeted therapy blocks the growth, proliferation or survival of tumor cells by inhibiting the correlated signal molecules (e.g., tyrosine kinase inhibitors, TKIs) rather than by cytotoxicity with traditional chemotherapy. However, TKIs such as sunitinib are only partially effective for mRCC. The resistance of targeted therapy includes adaptive resistance, intrinsic resistance and acquired resistance [3]. The tumor heterogeneity, dynamic variation and crosstalk of numerous cell death-related signaling pathways may be associated with the resistance of targeted therapy [4, 5]. Strategies to overcome drug resistance, to identify useful clinical prognostic markers and to predict the risk of unacceptable toxicity are urgently needed. In addition to targeted therapy, immunotherapy, such as immune checkpoint inhibitors that could activate the processes of T cell-induced cell death, was also explored and applied to mRCC treatment. Recent developments in various molecules are emerging as promising therapeutics for RCC, but all of the above strategies are ultimately more or less correlated with the processes of cell death in RCC.

## Targeted therapy for mRCC

To date, several subtypes of RCC have been defined, of which clear cell RCC (ccRCC) is the most frequent (75–80%), followed by papillary RCC (pRCC; 15%) and chromophobe RCC (chRCC; 5%) [6], and biallelic von-Hippel Lindau (VHL) gene defects occur in approximately 75% of sporadic ccRCC [7]. As the most common subtype of RCC, which accounts for most RCC-related deaths [8], ccRCC is frequently characterized by near-universal loss of the short arm of chromosome 3 [9], deleting several tumor suppressor genes. The key genetic inactivations or mutations for RCC include those in MET proto-oncogene (MET), polybromo 1 (PBRM1), transcription factor binding to IGHM enhancer 3 (TFE3), folliculin (FLCN), Tuberous Sclerosis Complex 1 (TSC1), fumarate hydratase (FH), succinate dehydrogenase complex subunit D (SDHD), phosphatase and tensin homolog (PTEN) and VHL [10, 11], which leads to the accumulation of downstream oncogenic targets, such as HIFs [12]. ccRCC develops resistance to apoptosis by diverse mechanisms, including VHL mutations [13]. Various diagnostic, prognostic, treatment and predictive biomarkers associated with angiogenesis in RCC have been used, of which VHL and its downstream HIF/VEGF pathway have been well understood, and associated targeted therapy has also been developed.

### VHL and the HIF signaling pathway

As a tumor suppressor, VHL, which is located on chromosome 3p25 and encodes 214 amino acids, is one of the most important genes associated with ccRCC. The VHL protein (pVHL) can inhibit angiogenesis and tumor growth and affect the stability of hypoxic induction factors (HIFs). HIFs, which are important inducers in the process of RCC canceration, will further lead to the expression of downstream genes, including vascular endothelial growth factor (VEGF), platelet-derived growth factor (PDGF), and carbonic anhydrase IX (CAIX), which are involved in cell proliferation, angiogenesis and erythropoiesis. Some ccRCCs are HIF-2 independent, and HIF-2, as a target in ccRCC, sets the stage for biomarker-driven clinical trials [14].

The regulator of the serine biosynthesis pathway, phosphoglycerate dehydrogenase, is a candidate therapeutic target for the elimination of advanced or metastatic ccRCC resistance to HIF-2α antagonists [15]. SLC6A3, a dopamine transporter that can be induced by hypoxia in normal renal cells and influenced by HIF-2α in ccRCC, serves as a novel, highly specific biomarker for ccRCC [16]. CAIX, which is induced by hypoxia and regulated by HIF-1α and abundantly overexpressed in ccRCC tumor tissue but expressed at extremely low levels in normal renal tissue or other histological types of RCC, could be regarded as a ccRCC-specific marker and serve as a prognostic marker in RCC cells [17]. CAIX expression increases with sunitinib targeted therapy, and lower CAIX levels are associated with a poor prognosis and possible resistance in metastatic ccRCC [18]. Promisingly, acetazolamide derivatives can bind to CAIX on the surface of RCC cells and selectively send payloads to the specific site of disease, sparing normal organs [19]. The (99m)Tc-labeled acetazolamide conjugate selectively targets RCC in vivo and may allow imaging of tumors in the kidney and distant sites at earlier time points [20]. The CAIX-directed nanoplatform loaded with a new class of apoptosis inducers in combination with sorafenib can alleviate drug resistance in RCC [21].

### VEGF/VEGFR targeted therapy

As a downstream target of HIF, VEGF, which can promote vascular permeability, extracellular matrix degeneration, vascular endothelial cell migration, proliferation and angiogenesis, can be specifically combined with its high-affinity receptor (VEGF receptor, VEGFR), which is mainly divided into three classes: VEGFR1, VEGFR-2 and VEGFR-3. Various corresponding drugs of VEGF monoclonal antibodies (McAb) and VEGFR tyrosine kinase inhibitors (TKIs) have been approved for mRCC targeted therapy (mainly including sunitinib, pazopanib, sorafenib, axitinib, cabozantinib, lenvatinib, and bevacizumab), and their corresponding combination therapy strategies have also been developed (Table 1).

**Table 1:** Main approved drugs for mRCC therapy under certain circumstances

Classification of the therapy method	Target	Drugs
Chemotherapy	DNA or RNA synthesis	Gemcitabine, doxorubicin
Nonspecific immune cytokines	Nonspecific immune cytokines	IL-2, IFN-α
Targeted therapy	VEGF	Bevacizumab
	VEGFR-TKI (multitarget)	Sunitinib, pazopanib, sorafenib, cabozantinib, axitinib, lenvatinib
	mTOR	Temsirolimus, everolimus
Immune checkpoint inhibitors	PD-1, PD-L1, or CTLA-4	Nivolumab, pembrolizumab, ipilimumab, atezolizumab
Combination therapy		Nivolumab + ipilimumab
		IFN-α + bevacizumab
		Bevacizumab + erlotinib
		Bevacizumab + everolimus
		Lenvatinib + everolimus

According to the number of amino acids, VEGFs can be divided into five different subtypes, VEGF121, VEGF145, VEGF165, VEGF189 and VEGF206, among which VEGF165 is the main form of VEGF. However, although VEGF165 has the highest amplification abundance and has been commonly used in clinical and experimental studies, another endogenous splicing variant, VEGF165b, has been found to resist angiogenesis and inhibit RCC growth [22]. As VEGFR-TKIs, sunitinib and pazopanib are the approved drugs in first-line therapy for patients with favorable- or intermediate-risk ccRCC. However, there have been only a few cases of complete and sustained responses to sunitinib. The treatment of non-clear cell RCC remains controversial, but often VEGF and mammalian target of rapamycin (mTOR)-related inhibitors are used, extrapolating data from the use of these drugs in ccRCC [23]. In pRCC2, the combination therapy of sunitinib and ABCC2 (ABC transmitter) blockers has therapeutic potential [24]. The tumor stroma expression of phosphorylated VEGFR2 (i.e., activated) might be taken as a predictive biomarker for clinical outcome in sunitinib-treated RCC patients [25]. Compared with sorafenib treatment, sunitinib treatment benefited from high expression of CAIX, HIF-2α and CD31 together with low expression of VEGFR1 and PDGFRB [26].

In addition to inhibiting the VEGF/VEGFR pathway, mTOR pathway inhibitors (everolimus and temsirolimus) have also been used in RCC targeted therapy (Table 1). Continuous activation of HIFs is important for the development of RCC and the acquisition of resistance to antiangiogenic multikinase and mTOR inhibitors. For patients treated with inhibitors of VEGF or mTOR, molecular subgroups of PBRM1, BAP1, and KDM5C mutations might have predictive values for metastatic ccRCC [27]. Interestingly, the administration sequence of first-line sunitinib followed by second-line everolimus, rather than everolimus followed by sunitinib, was supported for the treatment of patients with mRCC [28]. The sensitivity of sunitinib for RCC prognosis may also be predicted by a five-gene (BIRC5, CD44, MUC1, TF, and CCL5) signature [29].

### Single-nucleotide polymorphisms (SNPs) and RCC targeted therapy

Single-nucleotide polymorphisms of various molecules may also be used as candidate biomarkers of prognostic and therapeutic regimens, especially in genes related to angiogenesis and TKI pharmacodynamics. Five VEGFR1 genotyped SNPs (rs9582036, rs9554320, rs9554316, rs7993418 and rs9513070) were analyzed, and rs9582036 CC carriers had a poorer progression free survival (PFS) and overall survival (OS) compared to AC/AA carriers and thus could serve as potential predictive biomarkers for metastatic ccRCC patients receiving sunitinib treatment [30]. The genetic variants and polymorphisms of CYP3A5 and ABCB1 were also defined as predictors of sunitinib toxicity and efficacy, respectively, in mRCC treatment [31].

The main approved drugs for mRCC therapy under certain circumstances are shown in Table 1 and include chemotherapy (gemcitabine and doxorubicin for sarcomatoid differentiation, collecting tube or medullary subtype of mRCC), nonspecific immune cytokines (e.g., IL-2, IFN-α), targeted therapy, immune checkpoint inhibitors, and combined therapy. Although current research on the relative specificity of biomarkers for RCC has made great progress, the prediction of the clinical benefits of patients is still restricted to some extent because of the great heterogeneity and individual differences existing in molecular targeted drug therapy represented by TKIs. Among many of these potential biomarkers, the more distinctive markers for the accuracy of prediction in clinical practice are still being screened.

## Cell death-related molecules for RCC targeted therapy

Genetic intra-tumor heterogeneity is remarkable in ccRCC, where its presence complicates identification and validation of biomarkers in advancing precision cancer therapeutics [32]. As the existance of intra-tumoral heterogeneity, the tumour might include various collection of cells containing distinct molecular signatures with differential levels of sensitivity to therapy [4]. Targeted therapy can induce the death of most tumor cells, but a small heterogeneous subclone will survive and drive these cells to be resistant to therapeutic drugs. Thus, intra-tumor heterogeneity, which is correlated to heterogeneous protein function, is responsible for the therapeutic resistance to the conventional chemotherapy and radiation, and may promote tumor adaptation and therapeutic failure through Darwinian selection [33]. Furthermore, cancer stem cells may also account for the tumor cell heterogeneity formation and give rise to resistance to conventional chemotherapy and targeted therapy [34].

**Fig. 1:**
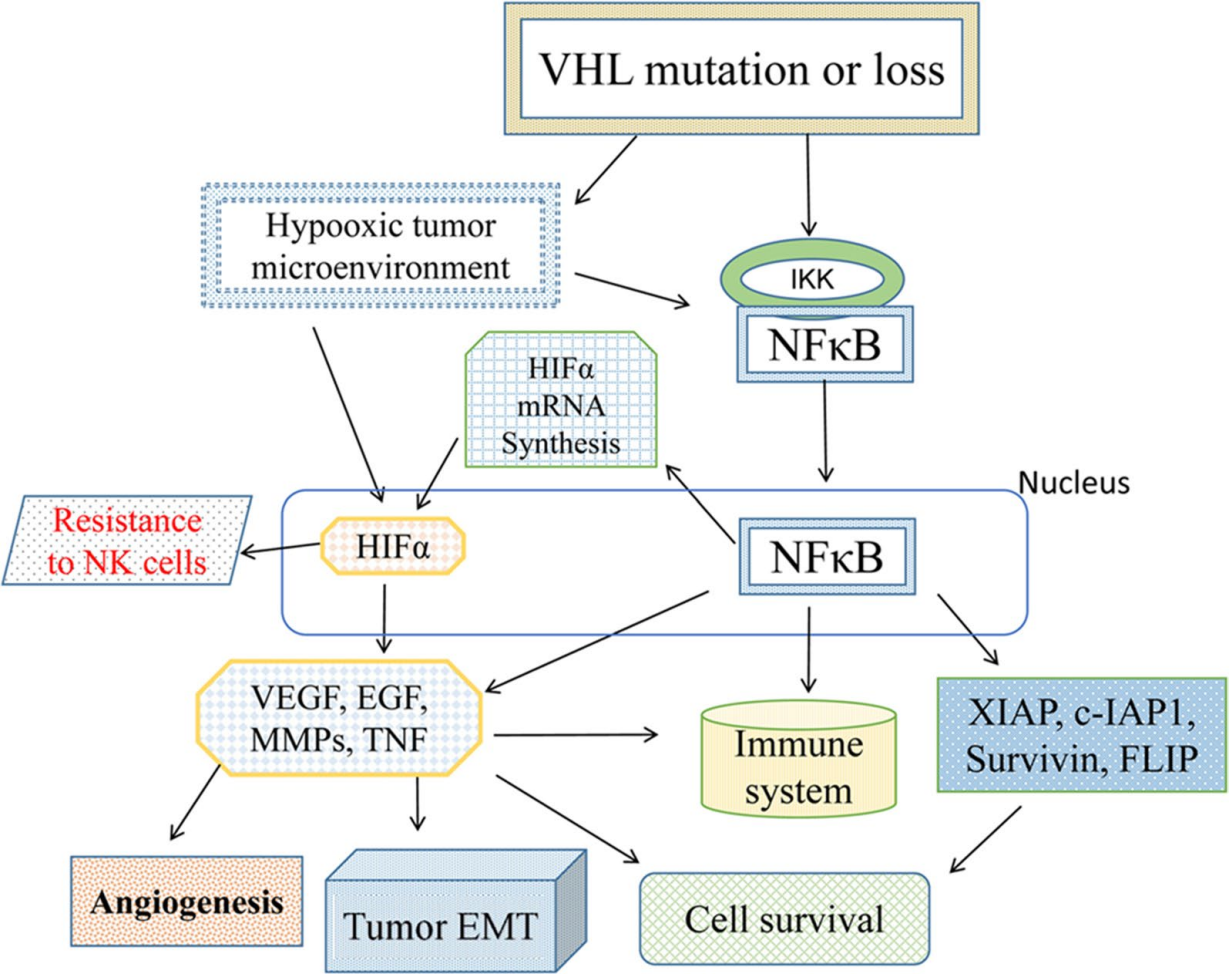
Interactions between VHL, HIF and the NF-κB signaling pathway. Inactivation of VHL initiates multiple downstream signaling pathways, including the HIF/VEGF and NF-κB transcription factor/HIF pathways, which become the basis of the crosstalk between cell death-related molecules, immunotherapy and targeted therapy

As the final stage, various forms of cell death that are induced by cytotoxicity either from exogenous or endogenous molecules and are modulated by multiple interconnected signaling pathways were originally defined and differentiated into apoptosis, necroptosis, pyroptosis, ferroptosis and autophagic cell death based on cellular morphology [35]. The tumor heterogeneity, dynamic variation and crosstalk of numerous cell death-related signaling pathways, such as phosphatidylinositol-4,5-bisphosphate 3-kinase (PI3K)/protein kinase B (AKT), mitogen activated protein kinases (MAPK)/extracellular regulated protein kinases (ERK), and inhibitor of NF-κB (IκB)/nuclear factor-kappa B (NF-κB), may be associated with the resistance of targeted therapy. NF-κB activation is a well-characterized consequence of the HIF-independent VHL deficiency signaling pathway [13, 36]. NF-κB essential modulator (NEMO)-driven VHL/HIF pathway activation is also involved in ccRCC progression [37]. As a well-known carcinogenic gene highly related to apoptosis, NF-κB, which also participates in necroptosis and autophagy, may serve as the key molecule associated with apoptosis, necroptosis, autophagy and the VHL/HIF pathway (Fig. 1).

### Apoptosis-related molecules for RCC targeted therapy

The evasion of apoptosis always occurs in cancer, and many molecules, such as programmed cell death 1 (PD-1)–programmed cell death 1 ligand 1 (PDL1)/PDL2, BCL-2, caspase and NF-κB, are associated with the processes of apoptosis. The apoptosis signaling transduction pathway can be divided into extrinsic (death receptor pathway), endogenous and endoplasmic reticulum (ER) stress-induced pathways according to the origin of the apoptosis signal and eventually converge on the activation of caspase. To date, only a small number of apoptosis-related small molecules have been found (Table 2). However, until recently, molecular drugs targeting the apoptosis-related pathway in RCC were rarely successfully developed.

**Table 2:** Apoptosis associated protein molecules and with RCC

Protein molecules	The function of the molecules	Targeted compound	The function of the compound on apoptosis
Bcl-2 family (Bcl-2, Bcl-xl, Bcl-w)	Anti-apoptosis	ABT-737, navitoclax, venetoclax, dicoumarol	Induce apoptosis
MDM2	MDM2-p53 interaction or MDM2-XIAP interaction	Nutlin-3, MX-69 [124]	Selective Mdm2 antagonist
IAPs (XIAP)	Inhibition of apoptosis	Embelin, Smac mimetic BV6	Inhibition of NF-κB pathway or sensitizes apoptosis
Caspase 3	Apoptotic executioners	Tasisulam	Caspase activator
PD-1	Immune checkpoint signal arrest (inhibit T cell induced cell death)	Nivolumab, pembrolizumab	Block the PD-1/PD-L1 or PD-1/PD-L2 interaction
PD-L1	Immune checkpoint signal arrest (inhibit T cell induced cell death)	Atezolizumab	Block the PD-1/PD-L1 interaction
NF-κB	A nuclear transcription factor drives the transcription of series anti-apoptosis genes in tumor cells	Curcumin, pyrrolidine dithiocarbamate, dexamethasone	Activication of Nrf2 pathway and inhibition of NF-κB activication
CTLA-4	Immune checkpoint signal arrest	Ipilimumab	Binds to CTLA-4 and blocks its interaction with its ligand (CD80/CD86)

### Extrinsic apoptosis pathway and targeted therapy

The tumor necrosis factor receptor (TNFR) superfamily serves as an important death receptor distributed on the cell membrane. Tumor necrosis factor-alpha (TNF-α), TNF-related apoptosis-inducing ligand (TRAIL) and anti-Fas induce death receptor-mediated extrinsic apoptosis by promoting the formation of death-inducing signaling complex (DISC) or TNFR1 complex II, resulting in caspase-8 and caspase-10 activation, and then cleave caspase-3 and caspase-7 to expand the death signal [38]. As a modulator of inflammation and the tumor environment, TNF-α not only activates the extrinsic apoptosis pathway but also gives rise to the activation of NF-κB, a transcription factor that regulates various apoptosis repressors; thus, the sensitivity of TNF-α to tumor cells shows a great difference. In addition, TNF-α has been used as a target of autoimmune disease, and the corresponding McAb was the number 1 selling drug in the first half of 2018 according to ranking data from the Ministry of Science and Technology of the People’s Republic of China (http://www.most.gov.cn/gnwkjdt/201809/t20180911_141661.htm) (Table 3).

**Table 3:** The global top ten sales of drug in the first half of 2018

No.	Drugs	Target	Category	Indications
1	Adalimumab	TNF	McAb	Autoimmune disease (rheumatoid arthritis; ankylosing spondylitis)
2	Apixaban	Factor Xa	Chemical drugs	For the prevention of venous thromboembolic events (VTE) in adults undergoing elective hip or knee replacement
3	Lenalidomide	Non-specific (immune regulation and anti-angiogenesis)	Chemical drugs	Multiple myeloma
4	Trastuzumab	HER2	McAb	Metastatic breast cancer and gastric cancer; adjuvant therapy of breast cancer
5	Rituximab	CD20	McAb	Therapy or combined therapy for specific type of non-hodgkin’s lymphoma
6	Etanercept	TNF	Fusion protein	Autoimmune disease (rheumatoid arthritis; ankylosing spondylitis)
7	Bevacizumab	VEGF	McAb	Combined therapy with chemotherapeutics for metastatic colorectal cancer and non-resectable advanced metastatic or recurrent non-squamous cell non-small cell lung cancer
8	Nivolumab	PD-1	McAb	Various specific type of advanced or metastatic cancers (melanoma; non-small cell lung cancer; renal cell carcinoma; head and neck squamous carcinoma; metastatic colorectal cancer; urothelial carcinoma; hepatoma) treated with corresponding anti-tumor drugs or containing corresponding biomarker
9	Pembrolizumab	PD-1	McAb	The treatment of an unresectable or metastatic melanoma that fails in first-line treatment
10	Infliximab	TNF	McAb	Autoimmune disease (active ankylosing spondylitis; ulcerative colitis, moderate or severe Crohn’s disease and fistula Crohn’s disease with poor conventional treatment; moderate or severe chronic plaque psoriasis and joint psoriasis; combined with methotrexate to treat moderate or severe active rheumatoid arthritis)

The ranking data was from the Ministry of science and technology of the People’s Republic of China (http://www.most.gov.cn/gnwkjdt/201809/t20180911_141661.htm)

Interferons (IFNs), toll-like receptors, TNF-α, TNFR1, and other possible mediators may also trigger signals to RIPK1 and RIPK3, both of which are required for necroptosis [39]. The activity of caspase-8 serves as a key regulator of TNF-induced apoptosis or necroptosis. In several cancers, TNF-α affected the epithelial-mesenchymal transition (EMT) and the expression of matrix metalloproteinase 9 and CD44, which may participate in the resistance of sunitinib therapy [40]. In fact, as one of the cancer stem cell marker, more than 20 isoforms of CD44 exists due to RNA alternative splicing, leading to different proteins in different cancer tissue subtypes [41]. High expression of CD44 in RCC correlates with high Fuhrman grade and recurrence, and serves as a poor prognostic marker for 5-year OS [42].

Preelevated TNF-α expression may make VHL-deficient cells more sensitive to cystine deprivation, which could induce necrosis [43]. The proteasome inhibitor bortezomib can enhance the sensitivity of TNF family death ligands to solid tumor apoptosis in RCC models [44].

Several apoptosis molecules and targeted combination therapies have been investigated, such as bevacizumab plus IFN-α [45]. Sorafenib can sensitize RCC cells to TRAIL-induced apoptosis not only by downregulating Mcl-1, a Bcl-2 family protein, but also by inducing reactive oxygen species (ROS) production, which might be a useful way to overcome TRAIL or other drug resistance [46]. Bortezomib pretreatment enhanced pro-caspase-8 activation and sensitized RCC to TRAIL-mediated apoptosis [47]. TNF and angiogenic or immunomodulatory mediators (e.g., interleukin-8, TGF-α, and VEGFR-2) were correlated with the risk of death, and they might be identified as markers of prognosis for benefit from VEGFR-TKIs in future studies [48]. The levels of soluble FasL (sFasL) in plasma and keratinocyte death mediated by the Fas/FasL interaction were significantly correlated with the hand-foot skin reaction caused by sunitinib [49].

#### Endoplasmic reticulum (ER) stress and RCC targeted therapy

Endoplasmic reticulum stress is characterized by an imbalance of calcium ions and the aggregation of misfolded and unfolded proteins inside the endoplasmic reticulum, which activates the type-1 ER transmembrane protein kinase, PKR-like ER kinase, and activating transcription factor 6 signaling pathways and directly influences the transformation of stress cells, such as adaptation, injury or caspase-12-mediated apoptosis. When ER stress is too strong or lasts too long and it is not sufficient to restore ER stability, it will eventually lead to apoptosis by an unfolded protein response.

VHL-mutant cells develop metabolic abnormalities that can cause chronic ER stress and the unfolded protein response [50]. The sunitinib-induced ER stress response induced by PERK may induce protumorigenic cytokine (IL-6, IL-8, and TNF-α) expression and contribute to sunitinib resistance in RCC patients [51]. Cuprous oxide nanoparticles can trigger ER stress-induced apoptosis and recover sunitinib responsiveness by regulating copper trafficking and by downregulating the expression of AXL, MET, AKT, and ERK in RCC cells [52].

### Necroptosis, autophagic cell death and ferroptosis in RCC targeted therapy

#### Necroptosis

**Fig. 2:**
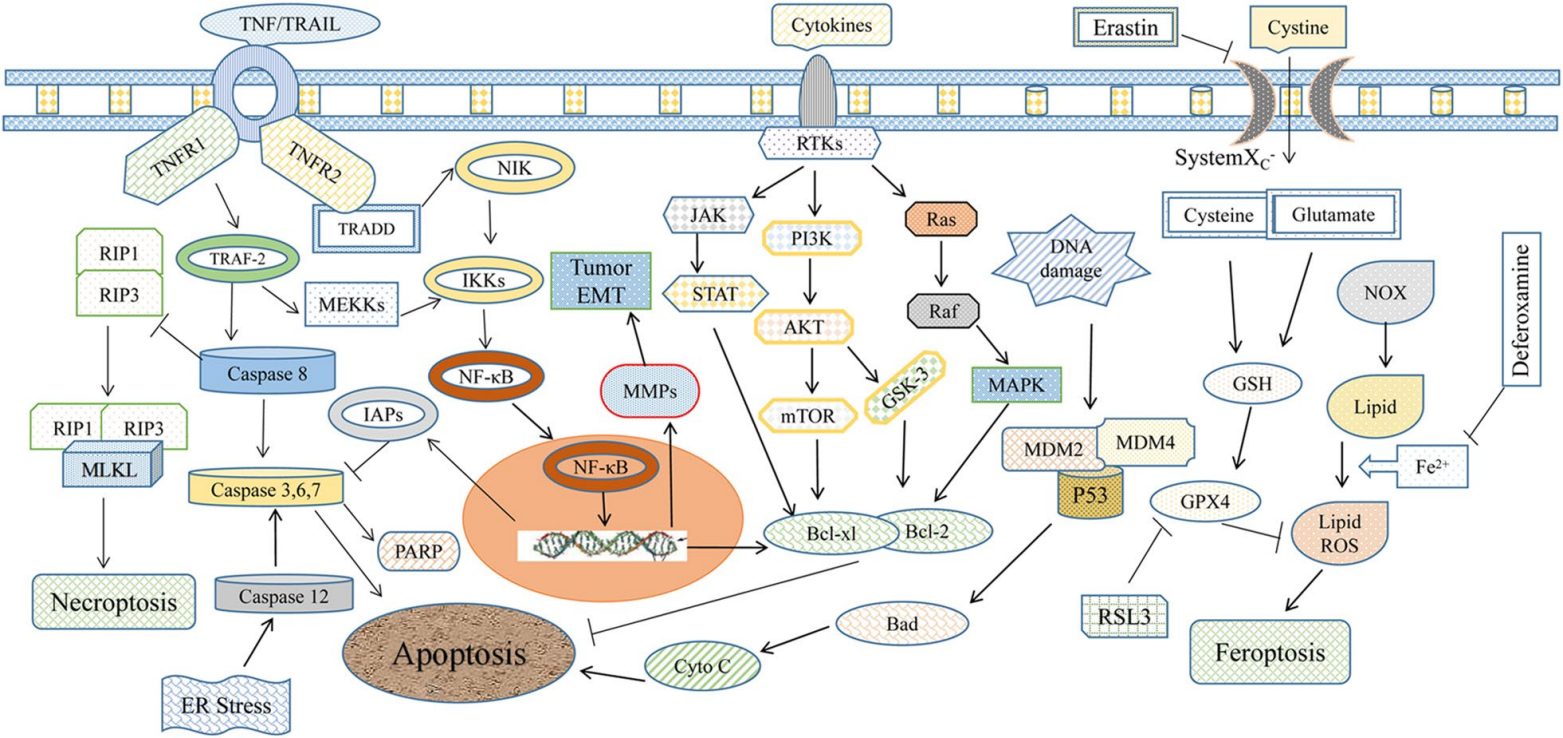
Apoptosis-, necroptosis- and ferroptosis-correlated signaling pathways. TNF/TRAIL initiates apoptosis/necroptosis signaling pathways depending on the activation or inhibition of caspase 8. Moreover, ferroptosis seems to be independent of apoptosis and necroptosis and is correlated with lipid ROS

In addition to apoptosis, a series of forms of cell death also exist in tumor progression (Fig. 2). Necroptosis is a caspase 8-independent inflammatory cell death or programmed form of necrosis, part of which has the same upstream signaling components as apoptosis. Necroptosis is mainly mediated by the activation of receptor interaction protein (RIP) 1, RIP3 and mixed lineage kinase domain-like (MLKL). TNF-α production leads to the stimulation of its receptor TNFR1, which can recruit many downstream molecules, such as TNFR-associated death protein (TRADD) and RIPK1, by transforming the polymer structure. The stimulated cells undergo apoptosis in the presence of caspase-8, but the cells will undergo necroptosis when caspase-8 is inhibited. Elevated RIPK1 and RIPK3 expression was observed in the most high-grade RCC cells, and either the apoptotic or necroptotic pathway was detected in response to TNF-α/TNFR1 signaling by using different inhibitors [53]. After prosurvival NF-κB signaling was inhibited by bortezomib, the resistant RCC cells were sensitized to necroptosis dependent on the RIP1 kinase triggered by IFN-γ [54].

#### Autophagic cell death and RCC targeted therapy

Autophagy and mTOR activation are considered survival mechanisms for RCC, and the protective autophagy is also involved in RCC therapeutic resistance. Autophagy-related stress tolerance can enhance cell survival by maintaining energy production that can give rise to tumor growth and therapeutic resistance. In addition, the feedback loops and crosstalks with other signaling pathways (i.e., PIM kinase family, PTEN expression, ERK/MAPK, Notch) are the underlying mechanisms involved in the acquired resistance to mTOR inhibitors [5]. The implication of translocation facor E3 and translocation facor EB in metabolic pathways and mTOR signaling are particularly intriguing in the processes of RCC tumorigenesis [55]. Correspondingly, mTOR inhibitors were developed as part of the current drugs for RCC therapy, as the biology of RCC is closely controlled by mTOR [56].

In tumor cells, accumulating evidence indicates that autophagy has a tight relationship with programmed cell death, while uncontrolled autophagy itself usually causes autophagic cell death [57]. Autophagic cell death is a type of cell death characterized by a large number of autophagosomes in organelles and the cytoplasm and differs from apoptosis and necrosis. As a VEGFR-TKI, sorafenib exerts its cytotoxic effect by inducing autophagic cell death in an Akt-dependent pathway instead of MAPK signaling [58]. However, in acquired sorafenib-resistant cells, ubenimex and 3-methyladenine can restore their sensitivity to sorafenib, indicating that autophagy participates in sorafenib resistance in RCC and that it can be reversed by processing of the Akt pathway [59]. STF-62247 induced autophagic cell death independent of HIF-1 in VHL-deficient cells, and its combination with radiation enhanced cell killing under oxic, hypoxic or physiological conditions [60].

#### Ferroptosis and RCC therapy

In addition to necroptosis, other types of cell death include iron-dependent ferroptosis, pyroptosis, anoikis, parthanatos, excitotoxicity, incorporation death (entosis), keratinization and so on [61]. Ferroptosis is a type of cell death that is different from apoptosis and autophagy; it is induced by iron-dependent oxidative damage, regulated by iron metabolism and lipid peroxidation signals, and marked by an increased cytoplasm, lipid ROS and enlarged mitochondrial membrane density (Fig. 2). ccRCC cells are highly dependent on β-oxidation and glutamine or cystine depletion in the processes of lipid peroxidation and ferroptosis [62]. After glutamine and cystine were converted into glutathione (GSH), lipid peroxidation and ferroptosis were inhibited (Fig. 2).

The deprivation of glutamine and cystine may represent an opportunity for RCC VHL/HIF-related therapy. In VHL-deficient cell lines and primary ccRCC cells, but not in VHL-restored counterparts, cystine deprivation induced rapid programmed necrosis [43]. The synthesis of GSH requires glutamine and cystine, and the inhibition of GSH synthesis by the deprivation of glutamine and cystine highly sensitized ccRCC cell growth in a MYC-dependent RCC mouse model [62].

### NF-κB-related molecules and RCC targeted therapy

#### NF-κB and RCC therapy

TNF-α, TRAIL and the FasL-mediated/TRAF2/NF-κB survival pathway can protect tumor cells from cell death. As a nuclear transcription factor, NF-κB, which consists of P50 (NF-kB1), P52 (NF-kB2), REL (also known as cREL), P65 (REL-A) and REL-B, drives the transcription of numerous genes associated with the resistance of apoptosis for tumor cells, such as inhibiting apoptosis inhibitors (IAPs), BCL-2, Bcl-xL, Cox-2, matrix metalloproteinases (MMPs), tumor necrosis factor (TNF) receptor-associated factors 1 and 2 (TRAF1, TRAF2), survivin, and XIAP.

Through non-canonical signaling via the upstream Tank binding kinase 1 (TBK1), fumarate could promote phosphorylation and accumulation of P65 at the HIF-1 promoter [63]. By downregulating NF-κB activity and its downstream (c-FLIP, survivin, c-IAP-1, and c-IAP-2) antiapoptotic proteins, pVHL promotes RCC cell cytotoxicity induced by TNF-α [64]. In RCC cells, sunitinib triggers a TRAF2-mediated NF-κB survival signaling pathway and a PERK-driven endoplasmic reticulum (ER) stress response, which may lead to resistance to sunitinib in RCC patients, and NF-κB inhibition restores the sensitivity of RCC cells to sunitinib [51]. TNF-α-induced NF-κB signaling in primary RCC cells or cell lines can be inhibited by IFN-α and IFN-γ [65].

NF-κB-mediated MUC13 promoted the growth and survival of RCC cells, while silencing MUC13 increased the killing effect of sorafenib and sunitinib to RCC cells and reversed their acquired resistance to these targeted therapy drugs [66]. The activation of oncogenic p21-activated kinase 1 (PAK1) identified a vital mechanism in RCC that maintained the stem-like phenotype and resistance to sunitinib by NF-κB/IL-6 activation [67]. As a member of the insulin-like growth factor binding protein family, insulin-like growth factor 2 mRNA binding protein 3 (IMP3) II, is an independent prognostic marker for localized ccRCC and has been validated to promote RCC cell migration and invasion by activating the NF-κB pathway [68].

#### Inhibitors of apoptosis protein (IAPs): survivin, c-IAP1 and the XIAP family

Inhibitors of apoptosis protein inhibit apoptosis by restraining caspase 3/7/9. Survivin expression was strongly associated with cancer progression in 273 patients with localized ccRCC [69]. High expression of survivin was associated with a poor prognosis and strong clinicopathological features in patients with RCC and could be used as a biomarker for RCC management [70]. An inhibitor of survivin, YM155, diminished and intercepted the transcription pathway of NF-κB and its target gene survivin, and a stimulator of NF-κB signaling, TNF-α, did not affect this type of inhibitory function. The combination of IFNα and Smac mimetic BV6 which antagonizes IAPs provides a promising strategy for synergistic induction of apoptosis in RCC cells [71].

#### MMPs and RCC

As a target regulated by the NF-κB transcription factor, MMPs belong to the proteolytic enzyme family, the members of which play a key role in tumor invasion and metastasis and can degrade various proteins in the extracellular matrix and destroy the cell tumor histologic barrier [72]. According to the role of the substrate and homologous fragments, MMPs can be divided into several groups as follows: collagenase, gelatinase, stromelysin, matrix degrading enzyme, furin activation of MMP and other secretion types of MMPs. By decreasing p52- and p65-DNA-binding activities, melatonin can transcriptionally inhibit MMP-9, and melatonin receptor 1A (MTNR1A)(high)/MMP-9(low) patients have a higher survival rate than MTNR1A(low)/MMP-9(high) RCC patients [73].

### BCL-2 family and RCC therapy

BCL-2 families can be divided into two kinds of proteins: antiapoptotic (Bcl-2, Bcl-xl, Bcl-w, McL-1, and CED9) and proapoptotic (Bax, Bak, Bcl-xs, Bad, Bik, Bid, etc.). Numerous small-molecule inhibitors of the BCL-2 family have recently been explored as novel antitumor therapeutic agents, such as ABT-737 and ABT-263. However, apoptosis induced by ABT-737 is often prevented by elevated Mcl-1 expression in several cancer cells. As a single agent, ABT-737 displayed little activity, but it potently killed RCC cells once antiapoptotic Mcl-1 was inhibited. For example, by downregulating Mcl-1 and upregulating Bim expression, cafestol is an example that can promote ABT-737 sensitivity to RCC cells [74]. With the existence of endogenous Noxa protein in RCC cells, the combination of chemotherapeutic drugs (such as etoposide or vinblastine) with ABT-737 can also overcome the protection from Mcl-1 and A1 [75].

Among numerous miRNAs, miR-15/16 can directly target BCL-2 and function as a tumor suppressor [76]. The cooperation of BCL-2 family members and apoptosis repressors with a CARD domain (ARC) will produce strong antiapoptotic effects, and targeting of ARC may be an important factor for therapeutic resistance and combination therapy strategies [1].

### p53 and MDM and RCC targeted therapy

As an important tumor suppressor, wild-type p53 regulates cell metabolism, cell cycle, cell senescence, apoptosis and drug resistance. Various mutations in p53 occur in tumors, and it is complicated to design effective targeted drugs specific to p53 mutations. Although wild-type p53 exists in most RCCs, its antitumor effects may be counteracted by variations in VHL, PBRM1, MDM2, MDM4, and HIF-1. Elevated expression of wild-type p53 is related to a poor outcome of RCC [77]. In RCC, six different isoforms of p53 have been reported, among which p53-β, by enhancing apoptosis in tumors, predicts a better prognosis of RCC patients [78].

Transglutaminase 2 (TGase 2) inhibition increases p53 stability, which synergizes with DNA-damaging drug (e.g., doxorubicin)-induced apoptosis, indicating that the combination of a TGase 2 inhibitor with a DNA-damaging agent may be a potential effective therapeutic approach for RCC [79]. Histone deacetylase 1 (HDAC1) inhibited the apoptosis-stimulating protein of p53-2 (ASPP2), but vorinostat, an HDAC1 inhibitor, restored ASPP2 transcription, elevated ASPP2, promoted apoptosis, inhibited the EMT and exerted a synergistic effect with 5-fluorouracil in vitro and in vivo in RCC models [80]. Angiopoietin-like protein 3 can bind to focal adhesion kinase and inhibit its nuclear translocation induced by sorafenib, attenuating p53 ubiquitination overexpression, enhancing the sensitivity of RCC cells to sorafenib and contributing to cellular apoptosis [81].

MDM2 and MDMX play a key role in p53 inhibition. An MDM2 antagonist, Nutlin-3, increases growth arrest and p53-dependent senescence in RCC cells, which is a strategy to rescue/enhance the antitumor function of p53 [77]. However, worse prognosis and low survival were displayed in patients with the MDM2 SNP309GG genotype, indicating that the polymorphism of MDM2 might be an independent poor prognostic factor for RCC [82]. In Caucasian female RCC patients, the homozygous G/G genotype of human MDM2 SNP309 is correlated with early onset [83].

### Caspase family and RCC therapy

Caspases, which act as both initiators and executioners of apoptosis, belong to a protease family with various members. In humans, 11 different caspases have been identified and can be divided into the caspase-1 subgroup (caspase-1, 4, 5, and 11), the caspase-2 subgroup (caspase-2 and 9), and the caspase-3 subgroup (caspase-3, 6, 7, 8, and 10). Caspase-1, 4, 5, and 11 are inflammatory-related caspases that are involved in pyroptosis. Caspase-3, caspase-6, and caspase-7 are the executioners of both endogenous and exogenous apoptosis. Caspase-8 is an important apoptosis regulator whose cell death-inducing activity is highly influenced by the insertion/deletion promoter polymorphism CASP8-652 6N ins/del (rs3834129), which may be correlated with the attenuation of the overall risk and metastasis risk of RCC [84].

The activation of caspase-8 lyses and inactivation of RIPK1 and RIPK3 form a complex with RIPK1 and FADD, triggering apoptosis, but the inhibition of caspase-8 derepresses RIPK1 and RIPK3, which interact with the RIP homology domain, and triggers necroptosis (Fig. 2). Englerin A (EA), a potent selective cytotoxic natural product against RCC cells, induced apoptosis in a caspase-independent manner and inhibited AKT and ERK activation but not the phosphorylation status of AMPK [85]. Physapubescin, a predominant steroidal lactone, can decrease HIF-2α expression and cause death receptor 5 (DR5) upregulation, caspase-8 and -3 activation, and cleavage of poly (ADP-ribose) polymerase (PARP), which serves as a novel proapoptotic agent targeting VHL-null RCC cells [86].

### Immune checkpoint and RCC therapy

Before the development of targeted therapy drugs, nonspecific immunotherapy drugs (cytokines: interleukin-2, IFN-α), which differ from immune checkpoint inhibitors, were used for the treatment of RCC. Until recently, in addition to targeted therapy drugs, inhibitors of the immune checkpoint have shown prospective antitumor activity and have been approved in a series of cancers, of which the PD-1 inhibitors nivolumab and pembrolizumab have entered the top ten global drug sales in the first half of 2018 (Table 3).

**Fig. 3:**
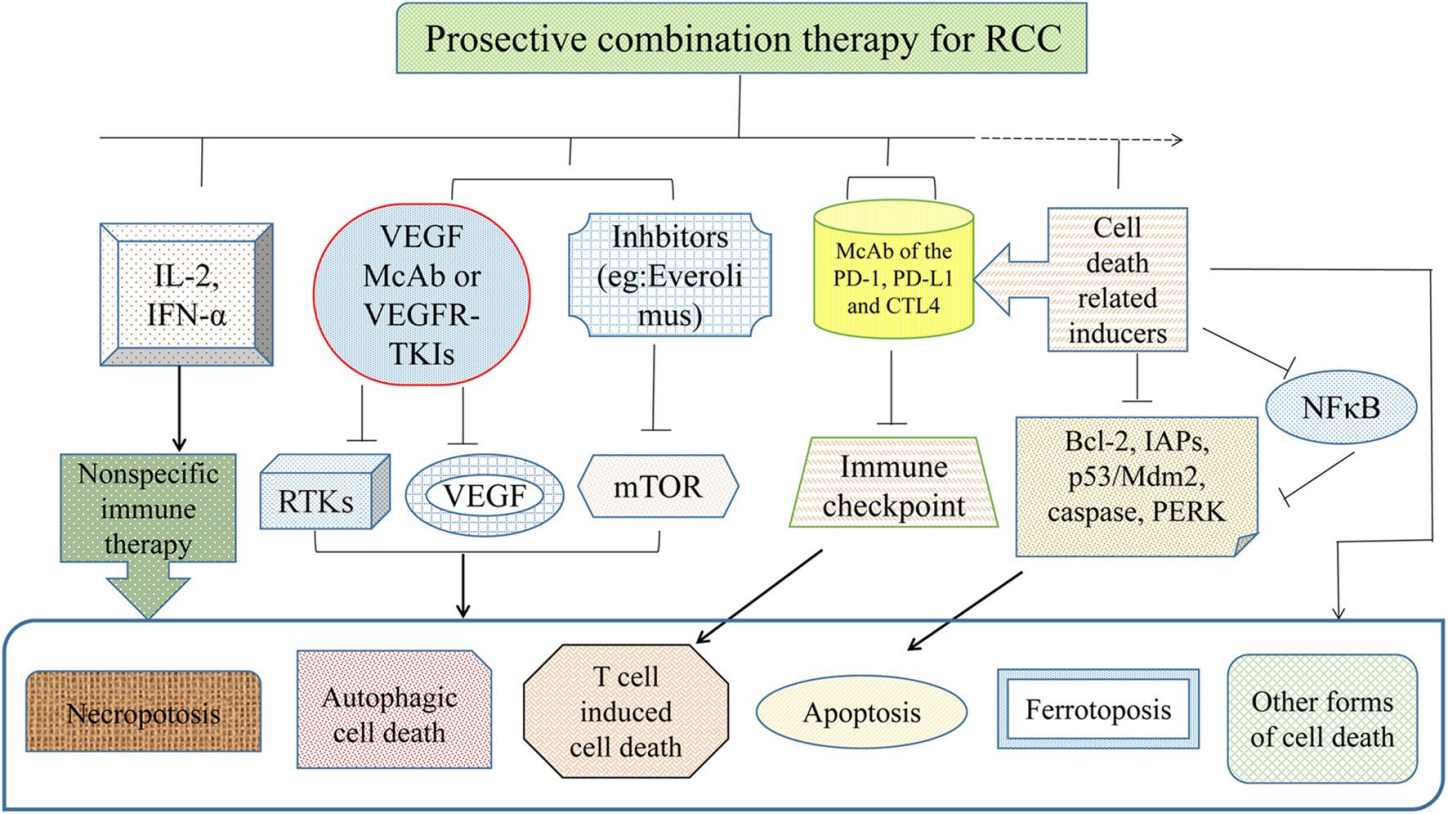
Prospective of combined therapy for RCC. Nonspecific immune therapy, targeted therapy, immune checkpoint therapy and novel new cell death-related molecules are shown, and their combination therapy is anticipated

In addition, the combination of targeted therapies, such as TKIs with immune checkpoint inhibitors, or the combination of immune checkpoint inhibitors is recommended or under investigation, and their combination with cell death-related signaling pathways is anticipated (Fig. 3). Among intermediate- and poor-risk patients with previously untreated advanced RCC, nivolumab plus ipilimumab displays higher OS and objective response rates (ORR) compared with those displayed by sunitinib in CheckMate 214 clinical trials. Particularly, as a new form of cell death, T cell-induced cell death by immune checkpoint inhibitors expands the treatment options beyond the current targeted therapy.

#### PD-1-PDL1/PDL2 and RCC therapy

PD-1-PDL1 plays a key role in T cell-induced cell death. PD-1 and its ligand PD-L1/PD-L2 plays a key role in tumor evading immune surveillance through negative immunomodulatory regulation. The combination of PD-1 and PD-L1 sends inhibitory signals to T and B cells by regulating the activation of effector T cells and ultimately weakens the antitumor immune response. Immune checkpoint inhibitors, such as PD-1/PDL-1 and cytotoxic T-lymphocyte-associated antigen (CTLA-4) blockade therapies, have led to considerable changes in the treatment of several solid malignancies, including metastatic melanoma, advanced RCC (aRCC), and NSCLC. In aRCC patients, checkpoint inhibitors, the PD-1 inhibitor nivolumab, the PD-L1 inhibitor atezolizumab and the CTLA-4 inhibitor ipilimumab alone or in combination with other agents are in various phases of clinical development [87].

The use of nivolumab as a new standard of treatment has been sustained across a wide range of patients with previously treated advanced RCC [88]. In ccRCC, the VHL mutation was positively correlated with PD-L1 expression and may influence the response of ccRCC to anti-PD-L1/PD-1 immunotherapy [89]. The expression of CTLA-4, PD-1, LAG-3, PD-L1, PD-L2, IDO1, and IL-10 were correlated with immunosuppression of the tumor microenvironment. Thus, these molecules should be considered high-priority targets in ccRCC patients for regulating immune responses, and the combination blockade of these molecules with immunotherapies might obtain synergistic antitumor activity [90].

The loss of PBRM1 in ccRCC may alter overall tumor cell expression profiles and affect its responsiveness to immune checkpoint therapy [91]. A PD-1 inhibitor combined with CIK cells led to strong antitumor activity in mRCC and NSCLC. The expression of PD-1 in tumor-infiltrating lymphocytes detected by immunohistochemistry, which is inferior to established prognosticating tools, is not related to the poor clinical outcome of ccRCC patients [92]. The percentage of coexpressing PD-1 and Tim-3 cells in tumor-infiltrating CD8^+^ T cells served as an important mediator, prognostic and predictive biomarker of aggressive phenotype, tumor size, risk of relapse and 36-month OS in RCC [93].

A retrospective analysis demonstrated objective responses and supported the use of nivolumab in heterogeneous non-clear cell RCC patients [94]. Heterogeneous intratumor PD1/PDL1 and HLA-G/ILT expression was found in both different areas of the same RCC and infiltrating immune cells, highlighting the necessity to fit personalized polyimmunotherapy [95]. In RCC, high expression of c-MET was correlated with lymph node metastases, sarcomatoid component, and overexpression of VEGFA and PD-L1 [96]. Interestingly, all wild-type VHL tumors expressed PD-L1, indicating that noninactivated VHL tumors and, in particular, wild-type VHL ccRCC are associated with PD-L1 expression, which could benefit from PD-L1/PD-1 blocking therapies [97].

As a HIF2-α target rather than a HIF1-α target, PD-L1, which is upregulated in pVHL-deficient ccRCC, may be an additional choice for ccRCC treatment with the combination of PD-L1 and HIF inhibitors [98]. Parenchymal polymorphonuclear myeloid-derived suppressor cells (PMN-MDSCs) were positively correlated with IL1β, IL8, CXCL5, and Mip-1alpha, while peripheral PMN-MDSCs were correlated with tumor grade. CXCR2 + PMN-MDSCs are critical for the decrease of anti-PD1 antibody activity, and anti-CXCR2 synergized with anti-PD1 in reducing tumor weight in an in vivo model [99].

#### CTLA-4 and targeted therapy

CTLA-4 molecules, which may be related to the occurrence and development of various malignant tumors, are involved in the negative regulation of the immune response, and their inhibitors (e.g., ipilimumab) have been explored as immune checkpoint drugs. The clinical benefit in a cohort of 63 ccRCC patients treated with PD-1 or PD-L1 inhibitor alone or combined with anti-CTLA-4 therapies was correlated with the loss of PBRM1, which may alter responsiveness upon immune checkpoint therapy [91]. Only partial patients respond to the pathways of immune checkpoint treatment, namely, CTLA-4 and PD-1/PD-L1, probably caused by profound immunosuppression, which may be partly induced by myeloid-derived suppressor cells (MDSCs), a potential predictive marker for the cancer therapy response [100]. Different immune microenvironments, such as mAbs of CTLA-4 and PD-1, have distinct immune-related adverse event (irAE) profiles and may induce histology-specific irAE patterns [101]. The G allele of CTLA-4 rs231775 showed a significant association with improved OS in metastatic ccRCC patients treated with sunitinib and might be used as a potential prognostic biomarker [102].

## Other independent molecules for RCC progression or targeted therapy

Ideal tumor biomarkers should have both specificity and high sensitivity and be secreted or present only in tumor tissue and specific to a certain type of tumor. Numerous molecules (e.g., GLI1, ENPP3, cytokeratin 7, KIM-1, SETD2, caveolin-1, miRNA, and lncRNA) were found to be diagnostic, therapeutic and prognostic markers for RCC [3, 103–105]. The histone methyl transferase EZH2, as an epigenetic modification target and marker, acted as a rational target and predictive marker for involved therapy in sunitinib-resistant ccRCC [106]. Recent developments in other small molecules targeting glutaminase, indoleamine-2,3-dioxygenase, C-X-C chemokine receptor 4, and TGase 2 are emerging as promising therapeutics for RCC [107].

The loss of PTEN is associated with tumor progression, including the occurrence and metastasis of RCC, and synergizes with sorafenib in inhibiting RCC cells [108, 109]. In RCC, a number of predicted microRNAs (miRNAs) are dysregulated and might participate in carcinogenesis, pathogenesis and aggressive tumor behavior. RCC-related tumor miRNA markers include miR-21 [110, 111], miR-132-3p [112], miR-141 [113], and miR-221 [114]. miRNA can also be regulated by long noncoding RNAs (lncRNAs), and the function of lncRNAs in cancer metabolism remains largely unexplored [115]. Until recently, a number of lncRNAs, including lncRP11-436H11.5 [116], lncMALAT1 [117], lncSARCC [118], lncRNA-HOTAIR [119], linc00152 [120], and lncARSR [121], were also found to be associated with RCC progression or targeted therapy. However, although SNP, epigenetic, miRNA, lncRNA and other molecular markers have been explored, many of these molecular markers also exist in other types of tumors and may not be specific to RCC.

## Future directions

As the existance of intra-tumoral heterogeneity, an accurate assessment of heterogeneity by all emerging technologies (i.e., multiregion sequencing, single-cell sequencing, analysis of autopsy samples, longitudinal analysis of liquid biopsy samples) for tumor-biopsy samples to dissect the complexity in the development of effective therapies and biomarkers for personalized-medicine will be anticipated [4]. On the other hand, as the the feedback loops and crosstalks with other numerous conventional cell death related pathways frequently occurred in targeted therapy, new form of cell death which is different from the past (i.e., T cell-induced cell death modulated by immune system) may have great potential for overcoming the resistance of targeted therapy. In addition, the combination of targeted therapy direct to the cancer stem cells with the conventional targeted therapy (i.e., TKIs) will also be prospective. Besides PD-1/PD-L1, more immune checkpoint or cell death related molecules (i.e., indoleamine 2, 3-dioxygenase, killer cell immunoglobulin-like receptor, lymphocyte-activation gene 3) will be discovered or revealed [122], and the combination of targeted therapy direct to the cancer stem cells with the immune checkpoint or cell death related molecules will also be promising for overcoming the multiresistance in RCC targeted therapy.

## Conclusions

The treatment for RCC has transformed over the past 12 years from a nonspecific immune approach (e.g., IL-2, IFN) to targeted therapy against VEGF/VEGFR and now to novel immunotherapy agents [123]. With the continuous development of targeted therapy and the rise of new immunotherapy drugs, the efficacy of advanced RCC has gradually improved. The sales of drugs targeting cell death-related molecules also yield significant benefits, and some of them ranked in the top 10 sales of the world in the first half of 2018 (Table 3). For targeted therapy, numerous strategies to overcome drug resistance and identify useful molecular markers have been found and discussed in a number of studies. However, multiresistance to targeted therapy frequently occurred because of the existence of heterogeneous subclones in RCC. As a form of cell death different from the past, T cell-induced cell death by immunotherapy drugs has great potential for overcoming the resistance of targeted therapy. Therapeutics of multiple targets associated with cell death may also be promising in RCC therapy [124]. In addition, the combination of targeted therapy, such as targeted therapy with immune checkpoint inhibitors, or the combination of immune checkpoint inhibitors is recommended or is under investigation [125]. The combination of cell death-related signaling pathways or molecular inducers with the above mentioned strategies (targeted therapy or immune checkpoint inhibitors) is also anticipated (Fig. 3). Instead of being expressed in normal kidney cells, more targeted therapies and cell death-related molecular markers specifically expressed in RCC are urgently needed. For targeted therapy, more specific features of each single RCC patient may need to be chosen and distinguished.

## Data Availability

The materials supporting the conclusions of this review are included in the article.

## Abbreviations

RCC: renal cell carcinoma
ccRCC: clear cell renal cell carcinoma
mRCC: metastatic renal cell carcinoma
pRCC: papillary renal cell carcinoma
MET: proto-oncogene MET
PBRM1: polybromo 1
TFE3: transcription factor binding to IGHM enhancer 3
FLCN: folliculin
TSC1: Tuberous Sclerosis Complex 1
FH: fumarate hydratase
SDHD: succinate dehydrogenase complex subunit D
PTEN: phosphatase and tensin homolog
VHL: von Hippel–Lindau
HIFs: hypoxia-inducible factors
EGF: epidermal growth factor
VEGF: vascular endothelial growth factor
PDGF: platelet-derived growth factor
TKIs: tyrosine kinase inhibitors
mTOR: mammalian target of rapamycin
PD-1: programmed cell death protein 1
PD-L1: programmed death ligand 1
VEGFR: vascular endothelial growth factor receptor
NSCLC: non-smallcell lung cancer
lncRNA: long non-coding RNA
IL: interleukin
PFS: progression-free survival
OS: overall survival
EMT: epithelial to mesenchymal transformation
BIM: Bcl-2 interacting mediator of cell death
ER: endoplasmic reticulum
CAIX: carbonic anhydrase IX
SNP: single-nucleotide polymorphisms
PI3K: phosphatidylinositol-4,5-bisphosphate 3-kinase
TBK1: Tank binding kinase 1
AKT: protein kinase B
NF-κB: nuclear factor-kappa B
NEMO: NF-κB essential modulator
TNF-α: tumor necrosis factor-alpha
TRAIL: TNF-related apoptosis-inducing ligand
IFNs: interferon
RIP: receptor interaction protein
DISC: death-inducing signaling complex
TRADD: TNFR-associated death protein
GSH: glutathione
ROS: reactive oxygen species
MMPs: matrix metalloproteinases
IMP3: insulin-like growth factor 2 mRNA binding protein 3
IAPs: inhibitors of apoptosis protein
FLIP: FLICE-like inhibitory protein
TGase 2: transglutaminase 2
ASPP2: apoptosis-stimulating protein of p53-2
HDAC: histone deacetylatlase
PARP: poly (ADP-ribose) polymerase
CTLA-4: cytotoxic T-lymphocyte associated antigen
PMN-MDSC: parenchymal polymorphonuclear myeloid-derived suppressor cells
McAb: monoclonal antibodies
MAPK: mitogen activated protein kinases
ERK: extracellular regulated protein kinases
